# Hierarchical Analysis of Physical Activity Determinants in Brazilian Adolescents: A Cross-Sectional Study

**DOI:** 10.3390/sports14010031

**Published:** 2026-01-08

**Authors:** Arley Santos Leão, Roberto Jerônimo dos Santos Silva, Naiara Ribeiro Almeida, Cinthya Luiza Rezende Oliveira, Diego Ignacio Valenzuela Pérez, Esteban Aedo-Muñoz, Ciro José Brito, Júlio Manuel Cardoso Martins, Aldo Matos da Costa

**Affiliations:** 1 Instituto Federal de Educação, Ciência e Tecnologia de Alagoas, Maceió 57020-600, Brazil; arleyleao@yahoo.com.br; 2 Department of Sport Sciences, University of Beira Interior, 6201-001 Covilhã, Portugal; jmartins4@gmail.com (J.M.C.M.); mcosta.aldo@gmail.com (A.M.d.C.); 3 Research Center in Sport Sciences, Health Sciences and Human Development (CIDESD), University of Beira Interior, 5001-801 Vila Real, Portugal; 4 Programa de Pós-Graduação em Educação Física, Universidade Federal de Sergipe, São Cristóvão 49100-000, Brazil; rjeronimoss@gmail.com; 5 Postgraduate Program of Physical Education, Federal University of Juiz de Fora, Juiz de Fora 36036-900, Brazil; naiara.ribeiro@ufjf.br (N.R.A.); cinthya.rezende@hotmail.com (C.L.R.O.); 6 Facultad de Salud y Ciencias Sociales, Universidad de las Américas, Santiago 8370003, Chile; 7 Escuela de Ciencias de la Actividad Física, el Deporte y la Salud, Facultad de Ciencias Médicas, Universidad de Santiago de Chile, Santiago 8380494, Chile; esteban.aedo@umce.cl

**Keywords:** physical activity, adolescents, hierarchical regression, substance use, weight perception

## Abstract

Background: Physical inactivity during adolescence remains a major public health concern, yet its multifactorial determinants are not fully understood in low- and middle-income settings. Objective: To identify and quantify the determinants of physical activity (PA) among Brazilian adolescents using a hierarchical logistic regression model that reflects the theoretical ordering of distal to proximal factors. Methods: A cross-sectional study was conducted with 856 adolescents (13–19 years). Data were obtained from the validated Brazilian Youth Risk Behavior Survey and the Brazilian Economic Classification Criteria (ABEP) socioeconomic questionnaire. PA was dichotomized according to World Health Organization recommendations. Hierarchical logistic regression examined five theoretical blocks: sociodemographic, anthropometric, substance use, weight/diet, and sedentary behavior. Results: Overall, 5 out of 17 predictors were significant in the full model. extended Body mass index (eBMI) was negatively associated with physical activity (OR = 0.331, 95% CI: 0.169–0.647, *p* = 0.001), while body mass was positively associated (OR = 2.078, 95% CI: 1.045–4.135, *p* = 0.037). Working status (OR = 1.235, 95% CI: 1.035–1.475, *p* = 0.019) and weight loss attempts (OR = 1.327, 95% CI: 1.042–1.690, *p* = 0.022) increased the odds of being active, whereas current smoking reduced it (OR = 0.715, 95% CI: 0.517–0.990, *p* = 0.043). Model discrimination improved progressively (AUC = 0.577 to 0.692). Conclusions: Physical activity among Brazilian adolescents was primarily influenced by behavioral and perceptual rather than demographic factors. These findings highlight the need for integrated interventions promoting muscle development, body positivity, and smoking prevention.

## 1. Introduction

Healthy habits established during adolescence, such as regular physical activity (PA), balanced nutrition, avoiding substance use, and maintaining an adequate body weight are strongly linked to better physical and mental well-being outcomes in adulthood [1,2]. Research shows that adolescents who engage in low-risk, health-promoting lifestyles tend to experience better self-rated well-being and decrease cardiovascular risk as adults, while those with high-risk behaviors face worse health outcomes later in life [2,3]. Dietary and physical activity patterns formed during adolescence often persist into adulthood, meaning early positive lifestyle choices can have long-lasting effects, including reduced risk of chronic diseases [4,5]. However, studies also highlight that positive behaviors tend to decline as adolescents transition to young adulthood, with increased sedentary time and decreased vigorous activity, emphasizing the importance of sustained support and intervention during this period [4,6]. Social support from family and school, as well as positive self-concept, are important factors which help maintain health-enhancing routines habits through these transitions [7]. Overall, promoting and supporting well-being-oriented behavior habits in adolescence is crucial for establishing a foundation for lifelong health and quality of life [3,4,5].

Building upon this understanding of how well-being-oriented habits influence later outcomes, it is equally important to consider the opposite spectrum, which is the engagement in risk-laden behaviors during adolescence [8,9]. Recent studies have indicated that adolescent risk behaviors include activities such as substance use, risky sexual behavior, insufficient PA, and delinquency, and these behaviors often co-occur and cluster together [8,9,10]. Research distinguishes between two main types: reactive risk behaviors, which are impulsive and reward-driven, and reasoned risk behaviors, which are planned and strategic, with the latter linked to greater sensation seeking and future orientation [11]. The likelihood of engaging in multiple risk behaviors increases with age, minor self-control, decrease socioeconomic status, and exposure to parental or maternal risk behaviors, as well as certain mental health conditions [8,12]. Personality traits such as impulsiveness, sensitivity to reward, and inferior social anxiety, along with male gender, are associated with higher risk-taking, while social and environmental factors like family structure, school engagement, and peer influence also play significant roles [10,12]. While most research notably focuses on negative risks, some adolescents engage in constructive challenges, or positive risk-taking, socially acceptable and constructive challenges, which can be beneficial for development [13]. Understanding these patterns and their predictors is crucial for designing effective prevention and intervention strategies tailored to adolescents’ diverse backgrounds and needs [8,9,10,12].

Extending this discussion to the Brazilian context, regional studies provide valuable insights into how these behaviors manifest locally [14,15,16]. For example, research using data from the Brazilian Scholar Health Survey has shown significant regional differences in physical activity and sedentary behavior among adolescents, with the Northeast and North regions reporting lower physical activity levels and higher sedentary time compared to other regions, and notable disparities between public and private school students as well as between capital and interior cities [14]. In this context, some studies have investigated lifestyle patterns and risk behaviors among adolescents in northeastern Brazil, highlighting the prevalence and clustering of unfavorable daily habits in this population [15,16,17]. Research in Sergipe found that nearly half of adolescents exhibited sedentary behavior, which was closely linked to insufficient PA, high consumption of soft drinks, psychosocial stress, loneliness, and sleep problems [17]. Another study in the metropolitan region of Aracaju revealed that male adolescents over 16 years old from greater socioeconomic backgrounds were more likely to engage in multiple simultaneous risk behaviors, such as low physical activity, excessive screen time, alcohol use, smoking, and involvement in fights [15]. Additionally, research in Pernambuco identified distinct clusters of risk-prone behaviors, with some adolescents combining smoking, drinking, and poor oral hygiene, while others showed patterns of unhealthy diets and low fruit and vegetable intake [16]. These findings underscore the importance of understanding how risk behaviors co-occur and are influenced by social, economic, and psychological factors in northeastern Brazil. Such evidence is crucial for designing targeted interventions and public health policies which address the specific needs and vulnerabilities of adolescents in this region [15,16,17].

Given the scarcity of analytical approaches capable of capturing these complex, multi-level determinants, advanced statistical frameworks have become increasingly relevant [18]. The use of hierarchical or multilevel analysis to investigate the determinants of physical activity among adolescents is both important and original, especially in the context of northeastern Brazil, where research on this topic is limited [19,20,21]. Hierarchical analysis enables simultaneously examining factors at multiple levels, such as individual, interpersonal, school, and environmental influences, providing a more comprehensive understanding of what drives or hinders physical activity in this population [19,22]. This approach is grounded in the social-ecological model, which recognizes that adolescent behavior is shaped by a complex interplay of personal, social, and environmental factors, and enables researchers to identify which levels have the greatest impact and how they interact [20,23]. Previous studies using hierarchical or multilevel methods in other countries have shown that factors like self-efficacy, parental and peer support, and access to facilities all contribute to physical activity, but their relative importance can vary by context [19,20,21,23]. By applying this advanced methodology in Northeastern Brazil, the study can fill a significant research gap; however, it also generates evidence which can inform more targeted and effective interventions and policies tailored to the unique realities of adolescents in this region. This originality enhances the scientific value of the research and its potential to improve adolescent well-being outcomes through context-specific strategies.

Despite the recognized importance of physical activity during adolescence in Northeast Brazil, most previous studies have examined determinants in isolation or using conventional regression approaches that fail to account for the hierarchical nature of influences on health behavioral patterns [15,16,17]. Hierarchical modeling represents a methodologically rigorous approach that respects the theoretical ordering of determinants, allowing researchers to examine how distal factors (i.e., sociodemographic characteristics) influence physical activity both directly and through more proximal factors (i.e., lifestyle perceptions and behaviors) [19,20,22]. This approach is particularly valuable for identifying independent predictors at each level of influence while controlling for confounding from higher-order variables, thereby providing clearer targets for intervention. However, few studies to date have applied hierarchical analysis to comprehensively examine physical activity determinants in Brazilian adolescents, representing a significant gap in the literature [24,25].

Therefore, the present study aimed to identify the multifactorial determinants of physical activity level in Brazilian adolescents using a hierarchical logistic regression approach, progressively examining five theoretical blocks: (1) sociodemographic characteristics, (2) anthropometric variables (extended BMI–eBMI), (3) substance use behaviors, (4) weight perception and dietary habits, and (5) sedentary behaviors. We hypothesized that: (a) sociodemographic variables (particularly sex and work status) would show significant associations with physical activity; (b) anthropometric variables (especially eBMI) would remain significant after controlling for sociodemographic factors; (c) substance use behaviors would negatively predict physical activity independent of sociodemographic and anthropometric characteristics; (d) weight management behaviors would show positive associations with physical activity; and (e) the full hierarchical model would demonstrate superior discrimination compared to models containing only sociodemographic and anthropometric variables, highlighting the importance of behavioral and perceptual factors in understanding adolescent physical activity patterns.

## 2. Materials and Methods

### 2.1. Research Design

This cross-sectional, school-based study was conducted between October 2019 and March 2020 at the Federal Institute of Education, Science, and Technology of Alagoas (IFAL), Brazil. The study design and reporting followed the Strengthening the Reporting of Observational Studies in Epidemiology (STROBE) guidelines to ensure methodological transparency and reproducibility. The target population consisted of adolescents enrolled in the Integrated Technical High School Program (1st–3rd years, equivalent to U.S. grades 10–12). In turn, a three-stage cluster sampling strategy was implemented to ensure representativeness across academic and regional contexts. In the first stage, a convenience sample of 10 of the 16 total IFAL campuses was selected. Selection was based on two primary criteria: (1) logistical accessibility for the research team to conduct the two-day data collection protocol, and (2) the availability of adequate computer laboratory infrastructure (minimum of 30 functional computers) to support simultaneous online data collection for full classes. The 6 excluded campuses were primarily those in more remote locations or with insufficient computer laboratory facilities. While this introduces a potential selection bias, the 10 included campuses represent a diverse range of geographic locations (both urban and rural settings) and academic programs (industrial, agricultural, information technology, and health sciences). In the second stage, courses were stratified by field of study (industrial, agricultural, information technology, and health sciences), and proportional numbers of classes were randomly drawn within each stratum. In the third stage, all students present in the selected classes on the day of data collection were invited to participate.

All study procedures complied with the Declaration of Helsinki and were approved by the Research Ethics Committee of the Instituto Federal de Educação, Ciência e Tecnologia de Sergipe (CEP/IFS) under protocol number CAAE 17282719.8.0000.8042 and approval number 3,559,205 on 6 September 2019. Participation was voluntary, and written informed consent was obtained from parents or legal guardians, along with assent from the adolescents. This structured approach ensured that data were collected from a representative and ethically approved sample of adolescents, allowing for reliable inference about the hierarchical determinants of physical activity.

### 2.2. Participants

The eligible student population at the time of data collection included approximately 900 students distributed across 10 campuses. Sample size was estimated a priori using G*Power 3.1 for logistic regression, assuming a small-to-medium effect size (odds ratio = 1.5), α = 0.05, and 80% power [26]. Considering 17 predictors and a design effect of 1.5, the target sample was increased from 550 to approximately 825 to account for potential non-response and clustering effects. Of the 948 students enrolled in the selected classes, 29 (3.1%) were absent on the day of data collection and 32 (3.4%) declined to participate or did not provide the required consent/assent forms. A total of 887 students participated, and after excluding 31 who exceeded the upper age limit (19 years), 856 students (90.3% of total enrolled) were included in the final analysis.

To justify the design effect (DEFF) of 1.5 used in the sample size calculation, we calculated the intracluster correlation coefficient (ICC) for the primary outcome (physical activity level) with school grades serving as the clusters. The average cluster size was 285.3 students (range: 252–326 students across 3 grade levels). The resulting ICC was negligible and not statistically significant (ICC = −0.0001, F_2, 853_ = 0.96, *p* = 0.38), yielding a calculated DEFF of 0.96. This indicates that the use of a DEFF of 1.5 in the a priori sample size calculation was a conservative and appropriate choice, ensuring sufficient statistical power for the hierarchical logistic regression analysis.

**Figure 1:**
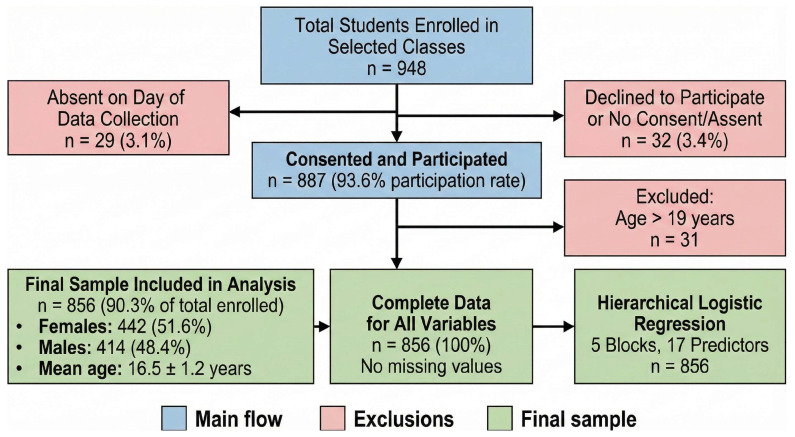
Participant flowchart detailing recruitment, exclusion criteria, and the final sample size included in the analysis.

Eligible participants were required to be enrolled in the Integrated Technical High School Program, aged 13–19 years, present in class on the data collection day, and to have submitted signed parental consent (for minors) and participant assent forms. Exclusion criteria included absence, lack of consent, age outside the specified range, or incomplete questionnaires. The final sample comprised 442 females (51.6%) and 414 males (48.4%), with a mean age of 16.5 ± 1.2 years. Participants represented all grade levels and course areas, providing a diverse and representative cross-section of IFAL’s adolescent population. Figure 1 shows the flowchart for selecting the participants.

### 2.3. Data Collection

Data collection occurred over two consecutive days at each campus following a standardized protocol. On the first day, researchers visited selected classes during regular school hours, explained the study objectives and ethical procedures, and distributed informed consent and assent forms. Students were instructed to return the signed documents the next day. On the second day, those who provided both forms completed the online questionnaire individually in the computer laboratory. Participants received standardized written instructions emphasizing confidentiality, voluntary participation, and the absence of right or wrong answers. The research team remained available for technical assistance but did not intervene during completion, which lasted approximately 25–45 min.

All researchers completed a four-hour training session on study procedures and ethics to ensure methodological consistency and data integrity. The questionnaire was pilot-tested with 30 students (excluded from the final sample) to identify and correct technical or semantic issues. Feedback from the pilot test led to three key modifications: (1) the addition of hover-over tooltips with concrete examples to clarify the distinction between moderate and vigorous physical activity (e.g., “brisk walking” vs. “running”); (2) debugging the questionnaire’s branching logic in the substance use section to ensure all conditional questions were displayed correctly; and (3) streamlining the socioeconomic questionnaire by removing three redundant items related to household appliances to reduce the average completion time from over 45 min to approximately 35 min, thereby mitigating response fatigue. Real-time validation minimized missing data and entry errors, and all responses were anonymous and completed on-site in a single session to reduce recall bias. Of the students approached, 887 submitted signed forms and completed the survey, yielding a final sample of 856 eligible participants after age-related exclusions.

### 2.4. Instruments

Data were collected using a composite online questionnaire integrating two validated instruments: the Brazilian version of the Youth Risk Behavior Survey (YRBS–Brazil) and the Brazilian Association of Research Companies (ABEP) socioeconomic classification questionnaire. The YRBS–Brazil, adapted and validated by Guedes and Lopes [27], assesses six categories of health-risk behaviors among adolescents, including behaviors linked to injury and violence, tobacco and substance use, sexual behavior, dietary practices, and physical inactivity. The instrument comprises six domains with the following structure: (1) behaviors contributing to unintentional injuries and violence (14 items), (2) tobacco use (6 items), (3) alcohol and other drug use (9 items), (4) sexual behaviors (8 items), (5) dietary behaviors (16 items), and (6) physical inactivity and sedentary behavior (8 items). For this study, items related to physical activity, sedentary behavior, substance use, and dietary habits were analyzed. For this study, selected items were used to assess PA levels, sedentary behavior, substance use, and dietary habits. PA was defined primarily based on sport and recreational contexts, consistent with the operationalization used in the YRBS–Brazil instrument. Work- or transport-related activities were not explicitly quantified. Participants were classified as physically active if they reported engaging in at least 60 min of moderate-to-vigorous PA on five or more days per week, in accordance with World Health Organization guidelines [28].

Self-reported height and weight were used to calculate eBMI = kg/m^2^, and although self-reported anthropometry may involve minor bias, previous research has demonstrated high validity (r > 0.90) among adolescents [29,30]. As an internal validity check, we analyzed the distribution of anthropometric data. The eBMI of our sample (mean = 21.9 ± 4.2 kg/m^2^, range = 13.2–58.8 kg/m^2^) was plausible, with only one extreme outlier (0.12% of the sample) identified and retained. Height ranged from 135 to 194 cm and body mass from 30 to 160 kg, with no implausible values detected. The prevalence of overweight/obesity in our sample (18.5%) was slightly shorter than national estimates for this age group (approximately 25–30%), which is likely attributable to the specific characteristics of the IFAL student population.

Socioeconomic status was assessed using the Brazilian Association of Research Companies (ABEP) questionnaire [31], which classifies households based on ownership of durable goods, education of the household head, and access to services. Participants were categorized into low, medium, or high socioeconomic levels according to total score. Additional demographic information, including sex, age, school year, and work status was collected via custom items appended to the questionnaire. Work status was assessed with a single, direct question: “Do you currently have a paid job?” with dichotomous response options (“Yes” or “No”). This question was designed to capture any form of formal or informal employment, including part-time jobs, apprenticeships, or internships, without specifying the type or intensity of work. The online format incorporated automatic response validation and branching logic, reducing missing data and optimizing data accuracy.

### 2.5. Statistical Analysis

All analyses were conducted in Python 3.11.0 using pandas, numpy, scipy, scikit-learn, matplotlib, and seaborn. Data were exported from Google Forms to Excel and imported into Python for processing. Initial procedures included data cleaning, range checks, outlier screening, and consistency verification. The final dataset included 19 variables with no missing values (n = 856), allowing complete-case analysis. Categorical variables were dichotomized based on theoretical and empirical criteria (see Table S1 for complete coding details). Physical activity level was classified as active (≥60 min of moderate-to-vigorous physical activity on ≥5 days/week) or inactive, following WHO guidelines. Substance use and dietary variables were coded ordinally (0 = none, 1 = once, 2 = ≥2 times). Continuous predictors were standardized (z-scores) to facilitate interpretation. Descriptive statistics were reported as means ± SD for continuous variables and frequencies (%) for categorical variables. Bivariate analyses included independent-samples *t*-tests and chi-square tests. Effect sizes were expressed as Cohen’s d and Cramér’s V, with 95% CIs. Pearson correlations examined collinearity, and multicollinearity was assessed using VIF (threshold < 5). All VIFs were acceptable. The main analysis used hierarchical logistic regression across five theoretical blocks: (1) Sociodemographic (sex, age, work status, economic level); (2) Anthropometric (body mass, height, eBMI); (3) Substance Use (smoking and alcohol-related variables, marijuana use); (4) Weight/Diet (weight perception, weight loss attempts, salad consumption); and (5) Sedentary Behavior (daily TV time). The complete hierarchical structure, including all variables in each block and detailed theoretical rationale for their ordering, is presented in Supplementary Table S2. The block order reflected assumed proximity to the outcome, from distal sociodemographic determinants to proximal behavioral factors. Model performance was evaluated using Nagelkerke R^2^, −2 log likelihood, likelihood ratio tests, ROC-AUC (95% CI via DeLong’s method), sensitivity, specificity, accuracy, and F1-score. Calibration was assessed with the Hosmer–Lemeshow test. Given the nested sampling design, intraclass correlation was examined and found negligible; therefore, standard logistic regression was used without cluster adjustments. Statistical significance was set at *p* < 0.05 (two-tailed), with interpretation emphasizing effect sizes and confidence intervals.

## 3. Results

The sociodemographic, anthropometric, and behavioral characteristics of the participants are presented in Table 1, stratified by physical activity level.

There were no significant differences in sex or age between active and inactive adolescents. Most participants were 16.5 ± 1.2 years old, and 92.1% reported having a job, those who worked were more likely to be physically active (*p* = 0.004). Economic level was not related to physical activity level. Inactive adolescents had slightly higher body mass (*p* = 0.008) and eBMI (*p* = 0.004) than active peers, though the differences were small. Height and television viewing time did not differ significantly between groups.

**Figure 2:**
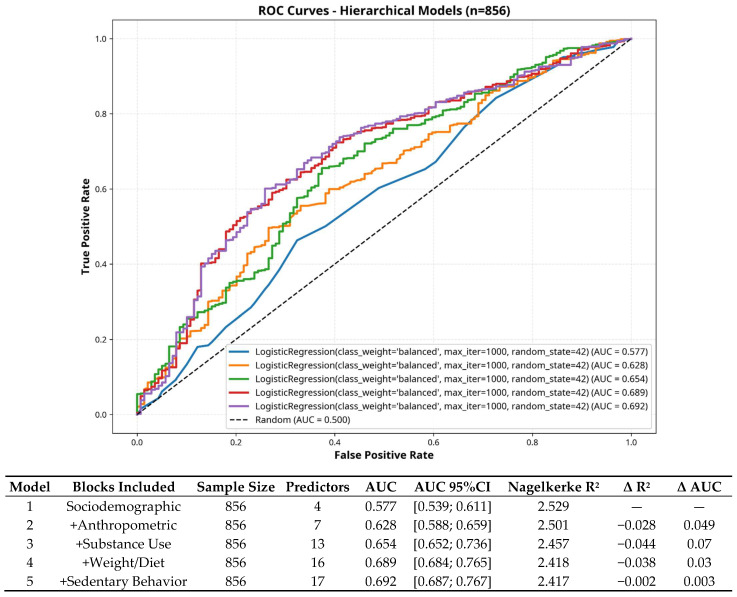
Five ROC curve models to predict the physical activity level. Blue line: sociodemographic, orange line: sociodemographic + anthropometric, green line: sociodemographic + anthropometric + substance use, red line: sociodemographic + anthropometric + substance use + weight/diet, purple line: sociodemographic + anthropometric + substance use + weight/diet + sedentarism. AUC—area under curve. CI—confidence interval; R^2^—coefficient of determination; Δ—delta.

Next, five hierarchical models were constructed for physical activity prediction by sequentially adding predictor blocks. Model 1, containing only sociodemographic variables, showed poor discrimination (AUC = 0.577). Adding anthropometric variables modestly improved accuracy (Model 2, AUC = 0.628). The inclusion of substance behaviors produced the largest gain (Model 3, AUC = 0.654), indicating that risk behaviors play a key role in predicting physical activity. Further additions of weight perception and dietary habits enhanced discrimination to AUC = 0.689 (Model 4), while the full model, including sedentary behavior, reached AUC = 0.692. Figure 2 shows the models constructed.

In the full hierarchical model (Model 5), 5 out of 17 predictors were statistically significant (*p* < 0.05). eBMI showed the strongest association (OR = 0.331, 95% CI: [0.169, 0.647], *p* = 0.001), with higher eBMI associated with substantially decrease odds of physical activity. Body mass paradoxically showed a positive association (OR = 2.078, 95% CI: [1.045, 4.135], *p* = 0.037), likely reflecting the contribution of muscle mass when controlling for eBMI and height. Work status was significant among sociodemographic factors (OR = 1.235, 95% CI: [1.035, 1.475], *p* = 0.019), with working adolescents 23.5% more likely to be active. Current smoking status showed a negative association (OR = 0.715, 95% CI: [0.517, 0.990], *p* = 0.043), while weight loss attempts were positively associated with activity (OR = 1.327, 95% CI: [1.042, 1.690], *p* = 0.022). Several variables that were hypothesized to predict physical activity did not reach statistical significance, including sex (*p* = 0.579), age (*p* = 0.121), alcohol consumption (*p* = 0.145), marijuana use (*p* = 0.111), salad consumption (*p* = 0.156), weight perception (*p* = 0.493), and TV viewing time (*p* = 0.419). Table 2 presents the complete regression results for Model 5.

## 4. Discussion

Current research often analyzes determinants in isolation, limiting the development of effective clinical and public health strategies. To address this gap, this study preformed a comprehensive hierarchical analysis of physical activity determinants in Brazilian adolescents, employing a methodologically rigorous approach that respects the theoretical ordering of influences posited by socio-ecological frameworks. Our primary objective was to move beyond conventional regression approaches by systematically examining how distal sociodemographic factors operate through more proximal behavioral and perceptual mechanisms to influence physical activity. This analysis allows us to synthesize the results in terms of direct effects versus mediating effects, as well as distinguishing between subjective versus objective factors. The hierarchical analysis with complete data yielded several novel and theoretically important findings. First, and most striking, anthropometric variables emerged as the strongest predictors of physical activity, with e-eBMI showing the most powerful negative association while body mass paradoxically showed a positive association when controlled for eBMI and height. This anthropometric paradox highlights the critical limitation of eBMI as a sole indicator of body composition and suggests that muscle mass, rather than overall adiposity, may be a key facilitator of physical activity in adolescence.

Second, work status, weight loss attempts, and current smoking emerged as significant independent predictors among behavioral factors, while other substance use behaviors (alcohol, marijuana) and dietary habits (salad consumption) did not remain significant after hierarchical adjustment, suggesting complex confounding and mediation patterns. Third, the progressive improvement in model discrimination validates the hierarchical approach and demonstrates that behavioral and perceptual factors add meaningful predictive value beyond demographic characteristics alone. These results have important implications for both etiological theory and intervention design, as they demonstrate that physical activity in adolescence is determined by a complex interplay between body composition, work context, weight management motivation, and substance use behaviors, rather than simple demographic or behavioral profiles.

The most striking finding of this study was the anthropometric paradox: eBMI emerged as the strongest negative predictor of physical activity, while body mass showed a paradoxical positive association when controlling for eBMI and height. The strong negative association between eBMI and physical activity is consistent with extensive literature documenting the inverse relationship between adiposity and physical activity in adolescents [32,33]. A greater eBMI, which primarily reflects greater fat mass, is associated with reduced physical capacity, increased perceived exertion, and potential weight-related stigma, all of which can discourage physical activity participation [34,35]. However, the positive association between body mass and physical activity, when eBMI and height are held constant, reveals a critical limitation of eBMI as a sole indicator of body composition [36]. This paradox likely reflects the fact that eBMI does not distinguish between fat mass and fat-free mass [36,37]. When eBMI and height are controlled, an increased body mass indicates greater muscle mass rather than adiposity [37].

Muscle mass is both a consequence and facilitator of physical activity: adolescents who are more physically active develop greater muscle mass, which enhances physical capacity and performance, creating a positive feedback loop [33]. This interpretation is consistent with research showing that physical self-concept and perception of physical competence, which are closely linked to muscle strength and motor skills, are stronger predictors of physical activity than BMI or body weight alone [38,39]. These findings underscore the critical need for more precise body composition assessment in physical activity research. While BMI is convenient and widely used, it conflates fat mass and muscle mass, potentially obscuring important relationships. From an intervention perspective, these results suggest that programs solely focused on reducing BMI or body weight may be less effective than those which promote muscle development, physical competence, and positive body image [38,40]. Strength training and skill-building activities that enhance physical self-concept may be particularly valuable for promoting sustained physical activity engagement among adolescents [39,41].

Weight management behaviors showed mixed results in the full hierarchical model. Adolescents attempting to lose weight were significantly more likely to be physically active, with 32.7% higher odds of activity. This finding supports evidence that exercise is a common and preferred weight-control strategy among youth, particularly compared to more restrictive dietary approaches [42,43]. The positive association between weight loss attempts and physical activity suggests that motivation for weight management can be leveraged to promote physical activity, provided that interventions emphasize health-oriented rather than appearance-focused messaging [40,44]. Thus, we point to the “body paradox”, distinguishing between the motivation for action to control the weight (cognitive-affective motivation) and how it is perceived by the actors themselves. However, weight perception itself was not a significant independent predictor of physical activity in the full model, despite theoretical expectations and some prior research showing that perceiving oneself as heavier or overweight predicts lower physical activity [34,35]. This insignificant finding may reflect complex mediation patterns: weight perception may influence physical activity primarily through its effects on weight loss attempts and body image, rather than exerting a direct independent effect. Alternatively, the relationship between weight perception and physical activity may be non-linear or moderated by other factors such as self-efficacy, social support, or weight stigma experiences [45,46].

These findings highlight the importance of distinguishing between weight management motivation (action-oriented) and weight perception (cognitive-affective). While motivation to lose weight can promote physical activity, negative self-perception and body dissatisfaction may inhibit it. Therefore, interventions should emphasize body positivity, health-oriented goals and skills development rather than messages focused on weight [40,45,46]. Programs that foster intrinsic motivation, perception of physical competence, and social support may be more effective in promoting sustained physical activity than those emphasizing appearance outcomes [38,39].

Substance use behaviors showed mixed results in the full hierarchical model. Current smoking status was the only substance use variable that remained a significant independent predictor of physical activity, with each increase in smoking frequency category associated with 28.5% lower odds of physical activity. This negative association is consistent with longitudinal studies showing reciprocal relationships between smoking and physical inactivity in adolescence [47,48]. Smoking may reduce physical activity through multiple mechanisms, including reduced cardiorespiratory fitness, increased perceived exertion during exercise, and clustering with other sedentary behaviors [48,49]. However, the consumption of other substances analyzed (alcohol and marijuana) has not produced statistically significant differences, which does not mean that they do not exist but may be mediated by other variables. This pattern suggests that the apparent effects of alcohol and marijuana use on physical activity may be confounded by other factors in the hierarchical structure, such as body composition, work status, or weight management behaviors. Of course, it will be necessary to continue with more precise and contextualized studies in a given society and moment [9,50]. The addition of substance variables notably produced the largest single improvement in model discrimination, confirming their collective explanatory role even though only one variable remained significant individually. This finding is consistent with evidence that substance use and physical inactivity tend to cluster as part of a broader pattern of health-risk behaviors in adolescence, shaped by overlapping risk factors such as sensation-seeking, small self-regulation, or competing time demands [9,51,52]. The clustering of health-risk behaviors has been reported across cultural contexts, suggesting that adolescents who engage in substance use are less likely to be active due to shared underlying determinants rather than direct causal effects of each specific substance [9,50].

These findings underscore the need for integrated public health strategies which address physical activity promotion and substance use prevention simultaneously, rather than treating them as isolated behaviors [49,53]. School-based programs that target multiple health behaviors together, emphasizing self-regulation skills, social support, and positive peer norms, may be more effective than single-behavior interventions [54]. Additionally, the finding that earlier smoking initiation (tried smoking, age started smoking) was not independently predictive suggests that current behavior is more relevant than historical exposure, reinforcing the importance of ongoing prevention and cessation efforts throughout adolescence [48,51].

Dietary and sedentary behaviors did not emerge as significant independent predictors of physical activity in the full hierarchical model. Salad consumption and TV viewing time were not significantly associated with physical activity after controlling for other variables. Eating patterns did not show significant differences, although we suggest some possibility of modifying the categorization processes; because the sensitivity of this study did not allow clear and realistic trends to be identified in relation to participants’ eating patterns [55]. The non-significance of salad consumption may reflect the complex and context-dependent nature of dietary behaviors. While healthy eating and physical activity are often conceptualized as complementary health-promoting behaviors, some adolescents may view dietary restriction and exercise as alternative rather than complementary weight-management strategies [56]. Salad consumption may additionally reflect weight loss intent rather than engagement in broader health-promoting behaviors, and its relationship with physical activity may be confounded by weight management motivation, which was directly measured and significant in this study [56]. Furthermore, the categorical coding of salad consumption (0 = none, 1 = once, 2 = two or more times) may also have limited sensitivity to detect associations. The non-significance of TV viewing time is particularly interesting given the prominent role of screen time in contemporary discussions of adolescent health [57,58]. However, this finding aligns with recent research questioning the assumption that sedentary behavior and physical activity are opposite ends of a single continuum, instead suggesting they are independent behaviors with distinct determinants [55].

Regarding sedentary behavior, a final factor to consider, of very high visibility in recent years, is the consumption of “screens”, although focused only on television this time. From the recorded data, no significant differences could be established between adolescents, with relatively low values (about 2 h per day), raising the hypothesis that young people have more leisure alternatives or use their free time in a more balanced way or with more diverse behaviors than we assume [55,56]. These findings highlight the complexity of adolescent health behaviors and underscore the importance of considering the broader social and behavioral context when designing interventions [51,56]. As explicitly noted, simple assumptions about “healthy” versus “unhealthy” behaviors may not capture the nuanced ways in which adolescents navigate competing demands, motivations and social norms; this will require better planning of future research in this field.

Work status was the only variable among sociodemographic factors which remained a significant independent predictor of physical activity, with working adolescents having 23.5% higher odds of being physically active compared to non-working peers. This association may reflect multiple mechanisms: the physical demands of certain jobs, the development of behavioral traits such as discipline, autonomy, and time management that favor an active lifestyle, or greater independence and mobility associated with employment [59]. Alternatively, the relationship may be bidirectional, with more physically active adolescents being more likely to seek and maintain employment due to increased energy levels and physical capacity.

Contrary to expectations and global trends, sex was not a significant predictor of physical activity in this sample. This null finding contrasts with most research showing that boys report greater physical activity levels than girls [60,61]. The absence of sex differences in this sample may reflect the specific context of technical education programs at federal institutes in Brazil, where academic focus, structured schedules, and institutional resources may attenuate gender-specific norms and opportunities that shape activity preferences in other settings. On the other hand, sex differences may be mediated by other variables in the model, such as work status, weight management behaviors, or body composition [62]. Age and economic level were also not significant predictors, possibly due to the relative homogeneity of this sample. The age range was narrow (13–19 years, mean 16.5 ± 1.2), and all participants were enrolled in the same educational system, which may have limited variability in these factors. Broader research consistently shows that older adolescents and those from smaller socioeconomic backgrounds are at greater risk for inactivity [60,61,62]. The non-significance of economic level in this sample may also reflect public educational policies in Brazil that provide relatively equitable access to sports and recreational resources across income groups within federal institutes, attenuating the socioeconomic gradient observed in other contexts. These findings reinforce that sociodemographic determinants of adolescent physical activity are context-dependent and should be interpreted within the framework of local cultural, educational, and structural conditions [59,61,62]. The relationship between socioeconomic status and physical activity is complex and can be moderated by gender, educational context, and policy environment [19,40]. Interventions should be tailored to specific populations and settings, considering the interplay of multiple sociodemographic factors and the availability of resources and opportunities for physical activity [59,61].

The progressive improvement in model discrimination from distal to proximal variables provides strong support for socioecological models of adolescent health behavior. Model discrimination improved systematically the AUC. This pattern validates the hierarchical approach and demonstrates that behavioral and perceptual factors add meaningful predictive value beyond demographic and anthropometric characteristics alone [63,64]. The largest single improvement in discrimination occurred when substance use behaviors were added (Model 2 → Model 3), confirming their collective explanatory role even though only current smoking remained significant individually in the full model. This finding is consistent with the concept of behavioral clustering or “syndemics”, where multiple health behaviors co-occur and are shaped by shared psychosocial determinants like self-regulation, sensation-seeking, and prevailing social norms [63,64,65]. The hierarchical approach allowed us to identify which variables exert independent effects after controlling for confounding, revealing that body composition, work status, weight loss attempts, and current smoking are the primary independent predictors, while other variables may operate through indirect pathways or shared underlying mechanisms.

From a theoretical perspective, these findings highlight the complex, multi-level nature of physical activity determinants in adolescence. Physical activity is not simply a function of individual choice or motivation but is embedded within a broader constellation of body composition, work context, weight management behaviors, and substance use patterns. The anthropometric paradox (BMI negative, body mass positive) underscores the importance of distinguishing between fat mass and muscle mass and suggests that physical capacity and physical self-concept may be more proximal determinants than body composition per se [33,41]. Furthermore, behavioral factors (work, weight loss attempts, smoking) remained significant after controlling for body composition suggests that these behaviors have effects on physical activity that are independent of their associations with body composition.

Limitations should be considered when interpreting these findings. First, the selection of campuses in the initial stage of sampling was based on convenience (accessibility and infrastructure), which may introduce selection bias and limit the generalizability of our findings to all 16 IFAL campuses. The 6 excluded campuses were primarily in more remote areas, and their student populations might have different characteristics. However, the 10 included campuses are geographically and academically diverse and represent the majority of the student population, mitigating this limitation to some extent. Second, the cross-sectional design precludes causal inference, and longitudinal studies are needed to confirm the directionality of the observed associations. Third, physical activity and other health behaviors were assessed via self-report, which may be subject to recall bias and social desirability bias. Future research should incorporate objective measures such as accelerometry to validate these findings. Fourth, while the calculated ICC was negligible, indicating minimal clustering effects, the use of school grades as the unit of clustering may not fully capture within-class or within-campus homogeneity. Fifth, although television viewing time was included as a sedentary behavior indicator, the questionnaire did not assess screen exposure from computers, smartphones, or tablets. This decision was based on the version of the YRBS–Brazil used, which included only TV-related items. The exclusion of broader screen-time measures represents a limitation, as contemporary adolescents are known to engage extensively with digital media across multiple devices [66]. Finally, the study was conducted in a specific educational context (federal technical high schools in Alagoas, Brazil), and generalization to other adolescent populations should be made with caution.

Despite these limitations, the large sample size, rigorous hierarchical design, and use of validated instruments strengthen the internal validity and relevance of the findings. Together, our findings advocate for integrated, multi-level programs that address physical activity [67], body composition, weight management, and substance use together, rather than treating them as isolated behaviors [53,54]. If we want our interventions in the beginning and in the adherence to the practice of physical activity to be successful, we must consider the cross effects between the indicated factors. School-based programs which combine physical activity promotion with body positivity messaging, strength training, smoking prevention, and support for working adolescents may be more effective than single-behavior interventions. Specifically, proposals for intervention should focus on the intrinsic motivation of participants, perception of physical competence; and the social support [38,39].

## 5. Conclusions

This hierarchical analysis of 856 Brazilian adolescents identified body composition as the strongest determinant of physical activity, with eBMI showing a powerful negative association and body mass paradoxically showing a positive association. This paradox highlights the limitations of solely using eBMI and suggests muscle mass facilitates activity. Work status, weight loss attempts, and smoking were also significant predictors. Model discrimination improved progressively, validating the hierarchical approach. These findings highlight the interplay between objective factors and subjective perceptions. Consequently, interventions should prioritize intrinsic motivation, perception of physical competence, and social support rather than solely focusing on weight reduction. Effective programs should integrate strength training, body positivity, smoking prevention, and support for working adolescents within comprehensive health promotion frameworks.

## Figures and Tables

**Table 1:** Sociodemographic and anthropometric characteristics of participants by physical activity level.

Variable	Inactive (n = 139)	Active (n = 717)	Statistic	*p*-Value	ES
Female	68 (48.9%)	374 (52.2%)	χ^2^ = 0.369	0.544	V = 0.021
Male	71 (51.1%)	343 (47.8%)			
Age (years)	16.6 ± 1.2 0.1 (−0.1; 0.33)	16.5 ± 1.1 0.1 (−0.11; 0.32)	t = 0.981	0.327	d = −0.091
Does not work	20 (14.4%)	48 (6.7%)	χ^2^ = 8.402	0.004 ^a^	V = 0.099
Works	119 (85.6%)	669 (93.3%)			
Low EC	73 (52.5%)	342 (47.7%)	χ^2^ = 1.597	0.45	V = 0.043
Medium EC	57 (41.0%)	310 (43.2%)			
High EC	9 (6.5%)	65 (9.1%)			
Body Mass (kg)	61.8 ± 9.6 2.91 (1.1; 4.8)	58.9 ± 12.2 2.91 (1.07; 4.76)	t = 2.65	0.008 ^a^	d = −0.246
Height (cm)	164.5 ± 3.7 0.05 (−0.7; 0.8)	164.5 ± 4.7 0.05 (−0.65; 0.76)	t = 0.129	0.898	d = −0.012
eBMI (kg/m^2^)	22.8 ± 3.6 1.1 (0.4; 1.8)	21.7 ± 4.3 1.1 (0.43; 1.78)	t = 2.868	0.004 ^a^	d = −0.266
TV Hours/Day	1.5 ± 1.7 −0.2 (−0.5; 0.14)	1.7 ± 1.9 −0.2 (−0.51; 0.12)	t = −1.142	0.254	d = 0.106

**Note:** data presented by mean ± standard deviation and 95% confidence interval. EC—economic level; TV—television; ES—effect size; ^a^ Statistically significant *p* ≤ 0.008 Inactive vs. Active. eBMI = extended Body Mass Index; d = Cohen’s d; V = Cramér’s V. Continuous variables were compared using independent samples *t*-tests. Categorical variables were compared using (χ^2^) chi-squared tests.

**Table 2:** Hierarchical logistic regression model 5, coefficients and odds ratios for physical activity level.

Variable	β	SE	OR	95% CI	Z	*p*
Block 1: sociodemographic						
Sex (Male)	−0.055	0.1	0.946	[0.778; 1.15]	−0.555	0.579
Age (years)	−0.182	0.117	0.834	[0.662; 1.049]	−1.55	0.121
Work Status (Yes)	0.211	0.009	1.235	[1.035; 1.475]	2.338	0.019 *
Economic Level	0.224	0.124	1.251	[0.981; 1.596]	1.802	0.072
Block 2: anthropometric						
Body Mass (kg)	0.732	0.351	2.078	[1.045; 4.135]	2.085	0.037 *
Height (cm)	−0.243	0.129	0.784	[0.608; 1.01]	−1.881	0.06
eBMI (kg/m^2^)	−1.106	0.342	0.331	[0.169; 0.647]	−3.234	0.001 **
Block 3: substance use						
Tried Smoking (Yes)	−0.025	0.182	0.976	[0.683; 1.394]	−0.136	0.892
Age Started Smoking	0.024	0.181	1.024	[0.718; 1.461]	0.131	0.896
Current Smoker (Yes)	−0.335	0.166	0.715	[0.517; 0.99]	−2.023	0.043 *
Age First Alcohol	0.035	0.104	1.035	[0.845; 1.268]	0.336	0.737
Alcohol Consumption (Yes)	−0.186	0.128	0.83	[0.647; 1.066]	−1.456	0.145
Marijuana Use (Yes)	0.208	0.131	1.231	[0.953; 1.59]	1.593	0.111
Block 4: weight/diet						
Weight Perception	0.101	0. 147	1.106	[0.829; 1.477]	0.685	0.493
Tried Weight Loss (Yes)	0.283	0.123	1.327	[1.042; 1.69]	2.297	0.022 *
Salad Consumption (Yes)	−0.172	0.122	0.842	[0.663; 1.068]	−1.419	0.156
Block 5: sedentary behavior						
TV Hours per Day	0.086	0.106	1.09	[0.885; 1.341]	0.808	0.419

**Note:** β = coefficient; SE = standard error; OR = odds ratio; CI = confidence interval; Z = z-statistic. * *p* < 0.05; ** *p* < 0.01. AUC = 0.692, Accuracy = 66.2%. Five significant predictors (*p* < 0.05).

## Data Availability

The original data presented in the study are openly available in FigShare at https://doi.org/10.6084/m9.figshare.30542603.

## Supplementary Materials

The following supporting information can be downloaded at: https://www.mdpi.com/article/10.3390/sports14010031/s1: Table S1. Variable Coding and Justification; Table S2. Hierarchical Block Structure and Theoretical Rationale.

## Abbreviations

The following abbreviations are used in this manuscript:

ABEP	Brazilian Association of Research Companies
AUC	Area Under the Curve
BMI	Body Mass Index
CEP/IFS	Research Ethics Committee of the Federal Institute of Sergipe
CI	Confidence Interval
EC	Economic Class
ES	Effect Size
IFAL	Federal Institute of Education, Science, and Technology of Alagoas
OR	Odds Ratio
ROC	Receiver Operating Characteristic
SE	Standard Error
STROBE	Strengthening the Reporting of Observational Studies in Epidemiology
TV	TV Hours/Day
V	Cramér’s V
VIF	Variance Inflation Factor
WHO	World Health Organization
