# Supplementary material for: Hierarchical Analysis of Physical Activity Determinants in Brazilian Adolescents: A Cross-Sectional Study
Source: Sports (Basel). 2026 Jan 8;14(1):31. doi: 10.3390/sports14010031 (PMC12846248; doi:10.3390/sports14010031)
Supplement: Supplementary file 1 [file sports-14-00031-s001.zip › sports-4004255-supplementary.pdf]

Table S1. Variable Coding and Justification.

Variable	Type	Coding	Cut-off / Categories	Justification
Physical Activity Level	Binary	0 = Inactive; 1 = Active	≥60 min MVPA on ≥5 days/week	WHO guidelines for adolescents (World Health Organization 2020)
Sex	Binary	0 = Female; 1 = Male	—	Natural binary variable
Age	Continuous	Years	13–19 years	Continuous to retain variability and detect linear trends
Work Status	Binary	0 = No; 1 = Yes	—	Natural binary variable
Economic Level	Ordinal	1 = Low; 2 = Medium; 3 = High	ABEP classification	Ordinal to capture socioeconomic gradient
Body Mass	Continuous	Kilograms	Self-reported	Continuous to retain variability and avoid information loss
Height	Continuous	Centimeters	Self-reported	Continuous to retain variability and avoid information loss
BMI	Continuous	kg/m^2	Calculated from $mass/height^2$	Continuous to detect non-linear associations with PA
Tried Smoking	Binary	0 = No; 1 = Yes	Ever tried cigarette smoking	Natural binary variable (lifetime exposure)
Age Started Smoking	Continuous	Years	Age at first cigarette	Continuous to detect age-related patterns
Current Smoker	Ordinal	0 = None; 1 = 1 time; 2 = 2+ times	Past 30 days	Ordinal to capture dose-response relationship
Age First Alcohol	Continuous	Years	Age at first full drink	Continuous to detect age-related patterns
Alcohol Consumption	Ordinal	0 = None; 1 = 1 time; 2 = 2+ times	Past 30 days	Ordinal to capture dose-response relationship
Marijuana Use	Ordinal	0 = None; 1 = 1 time; 2 = 2+ times	Past 30 days	Ordinal to capture dose-response relationship
Weight Perception	Ordinal	1 = Very underweight to 5 = Very overweight	Self-perception scale	Ordinal to capture perceptual gradient
Tried Weight Loss	Binary	0 = No; 1 = Yes	Past 12 months	Natural binary variable (recent behavior)
Salad Consumption	Ordinal	0 = None; 1 = 1 time; 2 = 2+ times	Past 7 days	Ordinal to capture dose-response relationship
TV Hours per Day	Continuous	Hours	Self-reported typical weekday	Continuous to detect linear and non-linear associations

Note: MVPA = moderate-to-vigorous physical activity; WHO = World Health Organization; ABEP = Brazilian Association of Research Companies; PA = physical activity.

Table S2. Hierarchical Block Structure and Theoretical Rationale

Block	Name	Variables	Theoretical Rationale
1	Sociodemographic	Sex, Age, Work Status, Economic Level	Most Distal Determinants: These fundamental demographic and socioeconomic characteristics shape opportunities, resources, and constraints for physical activity. They represent the broadest contextual factors that influence all subsequent levels. Sociodemographic factors determine access to facilities, time availability, and cultural norms around physical activity.
2	Anthropometric	Body Mass, Height, BMI	Intermediate Determinants: Body composition is partially determined by sociodemographic factors (e.g., socioeconomic status influences nutrition and growth patterns). In turn, body composition may influence behavioral choices related to physical activity through mechanisms such as perceived physical competence, comfort during movement, and body image concerns.
3	Substance Use	Tried Smoking, Age Started Smoking, Current Smoker, Age First Alcohol, Alcohol Consumption, Marijuana Use	General Behavioral Determinants: Health-risk behaviors tend to cluster together in adolescence, sharing common underlying determinants such as sensation-seeking, peer influence, parental monitoring, and self-regulation capacity. Physical inactivity is often part of this constellation of risk behaviors, reflecting a broader lifestyle pattern.
4	Weight/Diet	Weight Perception, Tried Weight Loss, Salad Consumption	Specific Behavioral/Perceptual Determinants: These factors are more proximal to physical activity, as they directly relate to energy balance, body image, and health consciousness. Weight-related perceptions and dietary habits are immediate precursors to physical activity motivation and behavior, often serving as triggers for initiating or maintaining active lifestyles.
5	Sedentary Behavior	TV Hours per Day	Most Proximal Determinant: Sedentary behavior directly competes with physical activity for discretionary time during waking hours. It represents the most immediate behavioral alternative to physical activity and is therefore the most proximal factor in the hierarchy. Time spent in sedentary pursuits (e.g., screen time) directly displaces opportunities for physical activity.

Note: This hierarchical structure is based on socio-ecological models of health behavior [1], which posit that behaviors are influenced by multiple levels of determinants, from distal contextual factors to proximal individual behaviors. The ordering from Block 1 (distal) to Block 5 (proximal) reflects the theoretical assumption that factors in earlier blocks influence those in later blocks, both directly and indirectly.

References

1. Golden, S.D.; Earp, J.A.L. Social ecological approaches to individuals and their contexts: twenty years of health education & behavior health promotion interventions. *Health education & behavior* **2012**, *39*, 364-372.
